# Oncolytic HSV: Underpinnings of Tumor Susceptibility

**DOI:** 10.3390/v13071408

**Published:** 2021-07-20

**Authors:** Chase Kangas, Eric Krawczyk, Bin He

**Affiliations:** Department of Microbiology and Immunology, College of Medicine, University of Illinois, Chicago, IL 60612, USA; ckangas2@uic.edu (C.K.); ekrawc4@uic.edu (E.K.)

**Keywords:** herpes simplex virus, oncolytic virus, virotherapy, interferon, STING, T-VEC, immunotherapy

## Abstract

Oncolytic herpes simplex virus (oHSV) is a therapeutic modality that has seen substantial success for the treatment of cancer, though much remains to be improved. Commonly attenuated through the deletion or alteration of the γ_1_34.5 neurovirulence gene, the basis for the success of oHSV relies in part on the malignant silencing of cellular pathways critical for limiting these viruses in healthy host tissue. However, only recently have the molecular mechanisms underlying the success of these treatments begun to emerge. Further clarification of these mechanisms can strengthen rational design approaches to develop the next generation of oHSV. Herein, we review our current understanding of the molecular basis for tumor susceptibility to γ_1_34.5-attenuated oHSV, with particular focus on the malignant suppression of nucleic acid sensing, along with strategies meant to improve the clinical efficacy of these therapeutic viruses.

## 1. Introduction

Cancer arises due to genetic or epigenetic changes that activate oncogenes, inactivate tumor-suppressor genes, or suppress innate immune genes. Although advantageous for proliferation or immune escape, such alterations often make cancer cells vulnerable to attack by oncolytic viruses [1]. To date, the most clinically advanced oncolytic virus is talimogene laherparepvec (T-VEC), which is a genetically engineered herpes simplex virus 1 (HSV) that has received international approval for the treatment of advanced melanoma [2,3,4]. T-VEC represents a significant milestone in the translational development of oncolytic immunotherapy and has inspired numerous and diverse oHSV platforms to emerge. Underlying the success of these oncolytic agents are common principles stemming from fundamental virus–host interactions exploited to destroy malignant tissue while leaving healthy tissue unharmed. In this review, we discuss recent progress in intracellular nucleic acid sensing machineries relevant to developing oncolytic viruses based on engineering of the γ_1_34.5 gene.

## 2. Selectively Engineered HSV as an Oncolytic Virus

**Figure 1:**
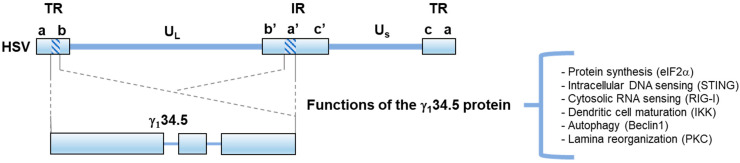
Schematic diagram of the HSV genome and the location of the γ_1_34.5 gene. The two covalently linked components of HSV-1 DNA, L and S, each consist of unique sequences, U_L_ and U_S_, respectively, flanked by inverted repeats (TR and IR). The reiterated sequences flanking U_L_, are designated as ab and b’a’, whereas the repeats flanking U_S_, are designated a’c’ and ca. The expanded portions beneath denote the γ_1_34.5 gene, which encodes a virulence factor with a large amino-terminal domain, a variable linker region, and a carboxyl-terminal domain. Known functions of γ_1_34.5 are outlined. The C-terminal domain of γ_1_34.5 mediates the dephosphorylation of host eIF2, preventing shut-off of protein synthesis. The N terminal domain of γ_1_34.5 targets STING and Beclin 1, inhibiting host DNA sensing and autophagy, respectively. Intact γ_1_34.5 is needed to modulate RIG-I, IKK, and PKC, inhibiting RNA sensing, DC maturation, and enabling laminar reorganization/viral egress.

HSV-1 is a large, enveloped DNA virus with a 150 kB length genome. The HSV genome is arranged in two regions of unique sequence (U_L_ and U_S_) flanked by two terminal repeated regions (TR) and one internal repeated region (IR) (Figure 1). Upon infection of mucosa, HSV will undergo lytic replication involving the sequential expression of three sets of viral genes: Immediate Early (α), Early (β), and Late (γ), leading to the production of infectious virions [5,6]. Following this, the virus will penetrate peripheral neurons to establish life-long latent infection, where HSV-1 undergoes periodic lytic reactivation in response to stress and other environmental factors [5,7].

The large genome and well-established biology of HSV-1 make it readily amendable to genetic manipulation for therapeutic use. A collection of effective antivirals, such as acyclovir, add an additional layer of safety on top of genetic attenuation. Several genes have been subjected to manipulation as the basis for the attenuation of oHSV. Among the most frequently modified is the γ_1_34.5 gene, which encodes an accessory factor required for neurovirulence [8,9]. HSV γ_1_34.5 is present in two copies per viral genome within the inverted repeat regions [10]. The γ_1_34.5 protein is multifunctional, with roles regulating protein synthesis, autophagy, nucleic acid sensing, and viral egress [11,12,13,14,15,16,17,18,19,20]. Importantly, these roles map to two functionally distinct domains, an amino-terminal domain and a carboxyl-terminal domain, which are connected by a triplet amino acid repeat linker region (Ala-Thr-Pro) (Figure 1). The triplet repeats are a constant feature of the γ_1_34.5 protein in HSV-1, but the number of repeats can vary among different strains [21]. Numerous studies have demonstrated the safety of both HSV deleted of γ_1_34.5 (Δγ_1_34.5) and γ_1_34.5-attenuated viruses at high doses of intracerebral inoculation in HSV-susceptible mice [8,9]. Additionally, to date, completed clinical trials involving γ_1_34.5-attentuated oncolytics ranging from Phase I to III demonstrate significant clinical benefits with limited adverse side effects (Table 1).

## 3. HSV Grapples with the Host Cytokine Response

HSV infection triggers the activation of host Pathogen Recognition Receptors (PRRs), which are responsible for initiating an antiviral response [22]. Among these PRRs are Toll-like receptors, which are transmembrane proteins on the cell surface and within endosomal compartments that detect the presence of extracellular Pathogen-Associated Molecular Patterns (PAMPs) such as LPS, dsDNA, dsRNA, and viral glycoproteins [23]. Additionally, the host has several intracellular nucleic acid sensors including RIG-I (RNA) [24] and cGAS (dsDNA) [25,26], among others. Activation of these sensing pathways converge on two critical proteins for initiating an anti-pathogen response, TANK Binding Kinase 1 (TBK-1), and Stimulator of Interferon Genes (STING). TBK-1 and STING will form a complex to activate IRF3 and IKK, which initiate Type I Interferon (IFNα/β) production and NF-κB signaling, respectively [22]. IFN and NF-κB will initiate the transcription of hundreds of genes to combat infection, attempting to create a non-permissive environment for pathogen replication while simultaneously secreting extracellular cytokines to direct the immune system toward the site of infection and initiate an adaptive immune response [27].

Wild-type HSV is capable of replication within an IFN-primed environment, and replication is severely restricted upon deletion of γ_1_34.5 [28]. As such, studies over the last decade have drawn light on how γ_1_34.5 overcomes IFN restriction at multiple levels of the immune response. At the level of nucleic acid sensing, γ_1_34.5 has been shown to directly bind and inhibit RIG-I [15]. Concurrently, γ_1_34.5 inhibits downstream adaptors, TBK-1 and STING, which are critical for innate immune signaling and IFN production [14,16]. Should IFN prime cells through upregulation of Interferon Stimulated Genes (ISGs) important for limiting HSV infection, such as dsRNA-dependent Protein Kinase (PKR), γ_1_34.5 and other viral proteins can block PKR and ISG activity to facilitate viral replication [28]. Additionally, γ_1_34.5 inhibits host autophagy, which is capable of degrading HSV capsids [13,29,30]. γ_1_34.5-mediated antagonism of TBK-1 and IKK in dendritic cells (DCs) results in impaired DC maturation, antigen presentation, and subsequent T lymphocyte activation [18]. Ultimately, γ_1_34.5 contributes to the creation of an immunosuppressed environment permissive to HSV replication. However, in the absence of functional γ_1_34.5, as is the case for many oHSV, unperturbed PKR, IFN, and NF-κB signaling can readily restrict replication, creating the need for additional accommodation to facilitate oncolytic replication.

## 4. Tumor Susceptibility to Δγ_1_34.5 oHSV Replication

Tumor susceptibility to Δγ_1_34.5 oHSV partially stems from the malignant suppression of antiviral pathways needed to limit replication in normal tissues [1]. Malignant antagonism of PKR was the first mechanism identified that could explain Δγ_1_34.5 oHSV replication in cancerous tissue [31]. In addition to PKR restriction, cGAS/STING DNA sensing is critical for limiting HSV infection, and recently, several studies have identified mechanisms in which cancer perturbs cGAS/STING signaling, providing an opportunity for Δγ_1_34.5 oncolytic replication [32,33,34,35].

### 4.1. PKR Pathway Regulation

**Figure 2:**
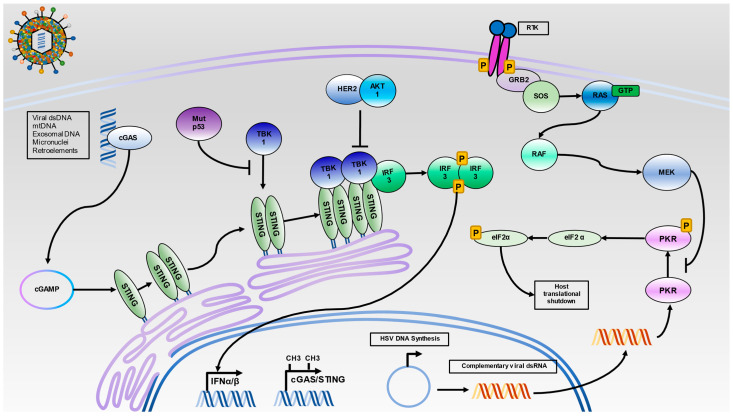
Intracellular Nucleic Acid Sensing by the STING and PKR Pathways. HSV infection results in the release of capsid into cells where viral dsDNA can activate cGAS. Additionally, dsDNA from several sources, including mitochondrial DNA (mtDNA), exosomal DNA, micronuclei, and reactivated retroelements can serve to activate cGAS. Once activated, cGAS will synthesize the cyclic dinucleotide c-GMP-AMP (cGAMP), which activates STING. Then, STING translocates from the endoplasmic reticulum to the Golgi apparatus. Oligomerized STING facilitates the trans-autophosphorylation of TBK-1 and subsequent recruitment of IRF3 to the STING signaling complex. The phosphorylation of IRF3 results in its dimerization and translocation to the nucleus for initiation of IFNα and IFNβ production. As infection progresses, viral DNA will circularize in the nucleus and undergo rolling circle replication. The onset of viral replication is thought to produce highly complementary viral dsRNA, which can activate PKR. The activation of PKR results in dimerization and trans-autophosphorylation. Then, activated PKR can phosphorylate eIF2α and shut down protein translation. In malignant cells, several mechanisms to silence intracellular nucleic acid sensing may operate. Hypermethylation of cGAS/STING promoters, inhibition of the TBK1-STING interaction by mutant p53, and TBK1 dissociation from STING by HER2 via AKT1 can prevent IRF3 activation. Additionally, unregulated Ras signaling can lead to constitutive activation of MEK1/2, which can inhibit PKR activation.

PKR is a dsRNA sensing serine threonine kinase constitutively expressed across a number of cell types that is upregulated in response to IFN stimulation [36]. PKR has several antiviral and anti-proliferative functions including shutdown of protein translation, activation of NF-κB signaling, activation of autophagy, and induction of apoptosis, making it a critical target for both virus and pre-malignant tissue [37]. Notably, PKR catalyzes the phosphorylation of eIF2α, which is a cellular protein required for the recycling of eIF2β. eIF2β carries the initial Met-tRNA needed for translation initiation. The phosphorylation of eIF2α at serine 51 greatly enhances eIF2α binding affinity for eIF2β, preventing GDP-GTP exchange factors from recycling eIF2β into the active GTP state needed for translation initiation [38]. Upon the detection of dsRNA, PKR will dimerize, inducing trans-autophosphorylation, which enables PKR to phosphorylate eIF2α at serine 51, resulting in shutdown of protein synthesis [36] (Figure 2).

The HSV genome contains many complementary transcriptional units thought to serve as a dsRNA source to activate PKR [39]. A major function ascribed to γ_1_34.5 pertains to its ability to maintain protein synthesis during late stages of infection [11]. This involves the C terminal domain of γ_1_34.5, which shares homology with host GADD34. The C terminal domain acts as a mediator to recruit both host Protein Phosphatase 1 α (PP1α) and eIF2α, facilitating the dephosphorylation of eIF2α by PP1α and preventing the shutdown of protein synthesis [12,40]. The importance of inhibition of PKR in HSV infection is evident in several studies showing that Δγ_1_34.5 replication and virulence are restored to wild-type levels in PKR −/− mice [41,42,43]. Although PKR’s status as a tumor suppressor is tenuous, showing both tumor suppressive and oncogenic activity [44,45], subsets of tumors are known to have perturbed PKR, providing an opportunity for Δγ_1_34.5 oncolytic replication in the absence of this restrictive antiviral factor.

Several known oncogenes and tumor suppressors influence PKR activity in the cancerous environment [31,37,46,47]. Approximately 19% of cancers exhibit Ras mutations, predominantly resulting in unregulated cellular proliferation [48]. Within this pathway, MEK, a MAPKK, has been shown to inhibit PKR activity [31]. Ras-transformation itself has been implicated to increase replication of wild-type HSV [49]. However, Smith et al. provide evidence in human cancer cell lines identifying tumor susceptibility to Δγ_1_34.5 oncolytic replication being correlated with MEK activity rather than Ras genotype in addition to being inversely correlated with PKR activation [31]. Pharmacological and genetic inhibition of MEK restores PKR expression and results in reduced Δγ_1_34.5 viral titers [31].

The IFN response is at least partially perturbed in 65–70% of cancers [1], underscoring the selection pressure to inhibit these pathways for tumor development. PKR is a critical ISG for Δγ_1_34.5 virus to overcome for efficient replication [28,41], indicating PKR has an important role in the story of malignant susceptibility to oHSV replication. Although the inhibition of IFN effectors such as PKR provides a precise method to facilitate viral replication or tumor progression, an alternative approach for HSV or pre-malignancy to achieve immune suppression is to target pathways responsible for initiating the IFN response and IFN production.

### 4.2. STING Pathway Regulation

Intracellular DNA sensing through cGAS/STING is a critical pathway for IFN production in response to both HSV infection and in pre-malignant environments [25,50]. Although the mechanism for HSV viral DNA exposure to the cytosol is unknown, HSV capsids can be degraded in a proteasome-dependent manner yielding cytosolic viral dsDNA for cGAS activation [51,52]. High concentrations of cytosolic dsDNA results in the release of cGAS from phosphatidylinositol 4,5 bisphosphate at the plasma membrane [53]. cGAS will dimerize and bind dsDNA in a sequence-independent manner [54]. Binding to long dsDNA results in optimal conformational changes for the production of cyclic GMP-AMP, a cyclic dinucleotide (CDN) capable of potently activating STING [55,56,57]. CDNs are commonly found as secondary messengers in bacteria and are also capable of activating STING [58]. CDNs bind to STING via its cytoplasmic domain and induce STING oligomerization and translocation from the endoplasmic reticulum to the perinuclear Golgi [59,60]. At the Golgi, STING undergoes post-translational modification, after which STING can recruit TBK-1 [61]. Oligomerized STING brings TBK-1 proteins into proximity, inducing the trans-autophosphorylation and activation of TBK-1. This allows for the recruitment and activation of IRF3, which will then dimerize and translocate to the nucleus to initiate Type I IFN production [62] (Figure 2). DNA sensing and subsequent IFN production places strong selection pressure on HSV to inhibit potent activation of the immune system. Several HSV proteins antagonize various steps of DNA sensing, from the degradation of cGAS itself (vhs) [63] to alteration of STING post-translational modifications (VP1-2) [64] to inhibition of STING translocation and TBK-1 binding (γ_1_34.5) [14,16], among other modes of inhibition [65,66,67,68]. Each of these virus–host interactions contribute to the inhibition of DNA sensing and facilitate successful HSV infection.

Notably, cancer exhibits a heavy reliance on genomic instability for tumor progression [69]. Genomic instability can lead to the formation of micronuclei prone to rupture, reactivation of endogenous retroelements, and cytosolic localization of mitochondrial DNA; each of these dsDNA sources can serve to activate cGAS/STING [70,71,72,73,74]. The activation of cGAS/STING DNA sensing is thought to be antitumorigenic, as the resulting IFN production can alert the immune system to identify and clear pre-malignant cells. Recent evidence has identified several mechanisms malignancy uses to perturb DNA sensing at multiple levels, including epigenetic silencing of cGAS/STING [33], mutant p53 inhibition of TBK-1 recruitment [34], and HER2 inhibition of STING signaling complex formation [35] (Figure 2).

DNA methylation, histone modifications, and non-coding RNAs represent epigenetic mechanisms commonly commandeered by cancer to alter its expression profile. DNA hypermethylation near promoter regions generally silences expression, whereas demethylated DNA is generally activating [75]. The hypermethylation of cGAS/STING promoters was initially implicated in a pair of studies analyzing the role of STING in colorectal cancer and melanoma cell lines [76,77]. Further in silico analysis revealed seven of 32 TCGA tumor types (bladder urothelial carcinoma, breast invasive carcinoma, colon adenocarcinoma, head and neck squamous cell carcinoma, lung adenocarcinoma, lung squamous carcinoma, and prostate adenocarcinoma) have significantly increased promoter methylation in either cGAS or STING as compared to matched normal tissue [33]. Interestingly, increased methylation of RIG-I and MDA5 genes was not seen in any of the 32 TCGA tumor types [33]. This is in line with previous studies indicating a majority of the melanoma and colorectal cancer cell lines analyzed have intact IFN production in response to poly IC dsRNA stimulation [76,77]. Recently, it has been identified that the use of demethylating agents can enhance cGAS/STING expression in silenced melanoma cell lines [78]. This reversal results in improved T lymphocyte recognition and increased IFN-γ production in an in vitro co-culture system [78]. Although much less prevalent (< 1% of tumors within TCGA database), it should be noted that several missense mutants of cGAS and STING were identified in the TCGA database [33]. In vitro reconstitution of mutant cGAS/STING in cGAS −/− or STING −/− MEF cells revealed that mutants had significantly reduced capacity to activate an IFNβ-luciferase promoter and show increased viral replication of Δγ_1_34.5 virus, further demonstrating the opportunity presented by cGAS/STING inhibition for oHSV [33].

DNA methylation contrasts with histone modifications as methyl moieties can be activating or inactivating depending on position and context [75]. Histone modifiers such as Lysine Demethylase 5 (KDM5) have been identified to inhibit STING expression in breast cancer [79]. Interestingly, another histone modifier, SUV39H1, is redirected by the Human Papilloma Virus oncogenic protein E7 to suppress STING expression [80]. Genetic and pharmacological inhibition of KDM5 or SUV39H1 results in increased STING expression and type I IFN production in breast cancer and HPV-transformed cells, respectively, in response to dsDNA treatment [79,80].

Widely known for its roles in apoptosis and tumor suppression, the transcription factor p53 is one of the most commonly mutated genes across cancer types: 47% ovarian, 43% colorectal, 40% head and neck, 38% lung, 35% melanoma, and 32% pancreatic [81,82]. Several in silico analyses have correlated p53 inactivation with immunosuppressed tumor phenotypes and decreased immune cell infiltration [83,84,85], but only recently has the role for mutant p53 in the suppression of cGAS–STING–TBK–IRF3 signaling been demonstrated [34]. Ghosh et al. found that nine forms of p53, which were mutated in either the DNA binding region or its structural domain region, were capable of binding TBK-1 in a cell transfection system. All nine p53 mutants reduced IRF3 activation by at least 60%, but three mutants show near complete abrogation of IRF3 activation. One mutant displaying near complete inhibition of IRF3 activation, p53R249S, was further characterized. The interaction between p53R249S and TBK-1 is sufficient to suppress IRF3-mediated apoptosis and IFN production in response to dsDNA stimulation. IFN reduction resulted in decreased CD4+ and CD8+ T lymphocyte infiltration in syngeneic mouse models of 4T1 breast cancer engineered with mutated p53R249S—as compared to wild-type p53 in non-modified 4T1. The extrinsic production of IFNβ was critical for the infiltration of tumors by CD4+ and CD8+ T lymphocytes. A lack of IFNβ, resultant from mutated p53R249S binding to TBK-1, leads to increased infiltration of immunosuppressive, M2 polarized macrophage in tumor tissues. Interestingly, mutant p53 interaction also blocks IFN production in response to poly IC and LPS stimulation, showing that the blockade of TBK-1 activity extends to other innate immune signaling pathways reliant on TBK-1 [34].

HER2 is a ligand-independent receptor tyrosine kinase. Upon activation, HER2 will homo/heterodimerize with other HER-family proteins at the cell surface, where it will then recruit and activate downstream effectors to influence cell proliferation and survival [86]. HER2 is mutated into a constitutively active form in ≈3.5% of cancers [87]. Recently, Wu et al. have identified HER2 as a potent inhibitor of STING signaling [35]. The intracellular domain of HER2 binds with the TBK-1-interacting C-terminal tail of STING. While this interaction is partially inhibitory itself, HER2 functions to recruit AKT1 to the STING signaling complex. AKT1 phosphorylates TBK-1 at serine 510, inhibiting TBK-1′s ability to bind in the STING complex, resulting in a significant decrease in IRF3 activation and subsequent production of ISGs. Importantly, constitutive STING signaling reduces tumor size in a mouse xenograft model using B16 melanoma, and tumor reduction is emulated when HER2 inhibitors are administered. Additionally, constitutively active STING increased CD4+ and CD8+ T lymphocyte infiltration, and the expression of HER2 significantly reduces infiltration in the B16 xenograft model [35].

## 5. Improving Tumor Selective Replication

While inhibition of cGAS/STING and PKR can provide an opportunity for Δγ_1_34.5 oncolytic replication, early evidence regarding first-generation oHSV such as HSV1716, R3616, and G207 show a wide range of replication efficiencies across tumor types [88,89,90,91]. The dichotomous role of PKR in tumor progression suggests that PKR is a barrier that must be overcome for efficient Δγ_1_34.5 oHSV replication in many tumor types [44,45], and studies characterizing cGAS/STING in melanoma, colorectal, and ovarian cancer cell lines have identified that epigenetic silencing, while prevalent and measurable, is not ubiquitous [76,77,92]. These studies have also indicated that activity of dsRNA sensing for the most part remains intact within these malignant cell lines [76,77,92]. Active PKR, TBK-1, cGAS/STING, and RIG-I all have the potential to restrict Δγ_1_34.5 replication [14,15,16,41], and the heterogenous inhibition of these pathways across cancer types provides a possible explanation for the replication variability observed with first-generation Δγ_1_34.5 oncolytics. Thus, several strategies have evolved from first-generation oHSV to enhance oncolytic replication and ultimately improve therapeutic efficacy (Table 1). Examined in further detail here are examples of oHSV that attempt to restore select functions of γ_1_34.5 to enhance oncolytic replication. The selective restoration of γ_1_34.5 has been the basis for using α expression of Us11, chimeric oncolytic HSV/HCMV hybrids, partial deletion of γ_1_34.5, and tumor-selective expression of γ_1_34.5 or γ_1_34.5 homologs to enhance oncolytic replication without restoring wild-type pathogenicity.

**Table 1:** Clinical stage oncolytic HSV based on γ_1_34.5 engineering.

Virus	Modifications	Clinical Trials	PMID	Reference
Seprehvir (HSV1716)	Deletion of two copies of γ_1_34.5, HSV-1 strain 17	NCT00931931, NCT01721018, NCT02031965	1848598	[9]
NV1020 (R7020)	Deletion of the HSV IR region (deletion of UL56, one copy of ICP0, ICP4, and γ_1_34.5), insertion of US2-2, US2-3, US2-4, and US2-5 derived from HSV-2	NCT00149396, NCT00012155	2842408	[93]
G207	Deletion of two copies of γ_1_34.5, insertion of lacZ into ICP6	NCT04482933, NCT03911388, NCT02457845, NCT00028158, NCT00157703	7585221	[94]
RP1	Deletion two copies of γ_1_34.5, deletion of ICP47 (α expression of Us11), insertion of GALV fusogenic protein; insertion of GM-CSF	NCT04050436, NCT03767348, NCT04349436	31399043	[95]
RP2	Deletion of two copies of γ_1_34.5, deletion of ICP47 (α expression of Us11), insertion of GALV fusogenic protein, insertion of anti-CTLA4 antibody	NCT04336241	31399043	[95]
RP3	Deletion of two copies of γ_1_34.5, deletion of ICP47, insertion of GALV fusogenic protein, insertion of proprietary stimulatory agents	NCT04735978	33072862	[96]
M032	Deletion of two copies of γ_1_34.5, expression of IL-12	NCT02062827	27314913	[97]
VG161	Deletion of two copies of γ_1_34.5; insertion of IL-12, IL-15, IL-15RA, and TF-Fc peptide capable of disrupting PD-1/PD-L1 interactions	NCT04758897, NCT04806464	33182232	[98]
ONCR-177	Deletion of the IR region, null ICP47 mutation; mutations in gB and UL37; insertion of miR-124-3p; insertion of miR-T cassettes targeting HSV-1 genes ICP4, ICP27, UL8, and γ_1_34.5; insertion of anti-CTLA4 (ipilimumab), FLT3LG, CCL4, IL12, IL12B, anti-PD-1	NCT04348916	33355229	[99]
talimogene laherparepvec (T-VEC)	Deletion of two copies of γ_1_34.5, deletion of ICP47 (α expression of Us11), insertion of GM-CSF	>50 Trials in clinicaltrials.gov, Select Trials: NCT00402025, NCT00289016, NCT00769704, NCT02263508, NCT02366195	12595888	[100]
OH2	HSV-2 backbone: Deletion of two copies of γ_1_34.5, ICP47 deletion (α expression of Us11), insertion of GM-CSF	NCT04637698, NCT04616443, NCT04386967, NCT03866525, NCT04755543	24671154	[101]
G47Δ	Deletion of two copies of γ_1_34.5, deletion of ICP6, deletion of ICP47 (α expression of Us11)	UMIN000002661, UMIN000010463, UMIN000011636, UMIN000034063	11353831	[102]
C134	Deletion of two copies of γ_1_34.5, insertion of HCMV IRS1	NCT03657576	15994764	[103]
rQNestin34.5v.2	Single copy of γ_1_34.5 under the nestin promoter	NCT03152318	32373649	[104]

### 5.1. α Expression of Us11

Us11 is a 21 kDa late-stage viral protein whose expression typically coincides with the onset of viral DNA replication [105]. Since their discovery, several functional overlaps between γ_1_34.5 and Us11 have been identified. Both exhibit inhibitory activity toward PKR [12,106], autophagy [13,107], RIG-I [15,108], and both contribute to full IFN resistance in late stages of HSV infection [109]. In hindsight, these overlaps make it clear why the serial evolution of Δγ_1_34.5 HSV in non-permissive glioma can select for α expression of Us11 [110,111]. α expression of Us11 can partially yet substantially restore viral replication without enhancing pathogenicity. As such, several oHSV use α expression of Us11 as a strategy to enhance oncolysis (Table 1).

T-VEC contains several modifications, including α expression of Us11 [100]. T-VEC has undergone clinical trials for the treatment of a myriad of solid tumors (Table 1). Approved as a standalone therapeutic for melanoma, many ongoing clinical trials use T-VEC in combination with either traditional cancer therapies (surgery and radiation) or with immune checkpoint blockade inhibitors [2]. G47Δ is an oHSV built upon the G207 backbone, containing deletions in both copies of γ_1_34.5 as well as the insertion of lacZ into the ICP6 gene along with the deletion of ICP47 yielding α expression of Us11 [102]. G47Δ shows enhanced in vitro replication and therapeutic efficacy in preclinical models as compared to G207, and this inspired several clinical trials in Japan for the treatment of glioblastoma, prostate cancer, olfactory neuroblastoma, and malignant mesothelioma (Table 1).

### 5.2. Chimeric oHSV

Another approach to improve oncolytic replication is to utilize non-native viral proteins functionally similar to γ_1_34.5 or proteins to perturb angiogenesis [112]. One such virus, C134, expresses IRS1, derived from Human Cytomegalovirus (HCMV). IRS1 is a dsRNA binding protein capable of binding to and blocking PKR-mediated shutdown of protein synthesis in the context of Δγ_1_34.5 infection [103,113] but is unable to prevent IRF3 signaling when expressed in the Δγ_1_34.5 backbone [114]. These functions serve to enhance the replication of C134 in several glioma models [115]. Preclinical testing of C134 in *Aotus nancymaae* demonstrated safety in this model [116], and in 2019, C134 entered Phase I clinical trials enrolling 24 patients for the treatment of glioblastoma multiforme set to complete in late 2024 (Table 1).

### 5.3. Tumor-Selective Expression of γ_1_34.5

Several oHSVs utilize tumor-selective promoters to express γ_1_34.5. The selective expression of γ_1_34.5 allows for increased viral spread and replication within transcriptionally targeted tissues. To date, these viruses have been developed using an oncolytic backbone deficient in both copies of γ_1_34.5; then, a single copy of γ_1_34.5 is reintroduced under tumor-selective promoters for proteins such as b-myb and nestin [117,118]. Nestin is an intermediate filament expressed specifically in glioma. Several oHSV use the nestin promoter, including rQNestinv.2, which is set to complete a Phase 1 clinical trial in July 2021 for the treatment of recurrent glioma. NG34 is a recent experimental oncolytic virus using the nestin promoter to selectively express host GADD34 [119]. GADD34 is homologous to the γ_1_34.5 C-terminal domain and functions in the cell to negatively regulate eIF2α kinases such as PKR. Interestingly, chemotherapy and radiation have been shown to upregulate GADD34, which specifically enhances therapeutic effect of other single deletion γ_1_34.5 oHSVs [120,121]. ONCR-177 is another oHSV that employs miRNA to selectively inhibit the expression of γ_1_34.5 in normal tissues and thereby enables tumor-selective viral replication [122]. In addition to tumor-selective expression of γ_1_34.5 itself, tumor selectivity can be achieved through an alteration of HSV tropism. Retargeting HSV to tumor biomarkers has an advantage through the selective expression of wild-type viral proteins such as γ_1_34.5, which can facilitate replication in targeted tissue. One example involves the alteration of oHSV entry proteins to target cancer cells expressing HER2 [123,124].

### 5.4. Partial Deletion of γ_1_34.5

Three decades of research have mapped numerous functions within the γ_1_34.5 protein. The TBK-1, STING, IKK, and Beclin 1 interaction regions all lie within the N-terminal domain of γ_1_34.5 [13,14,17,18]. ΔN146 is an experimental oncolytic virus in which the N-terminal domain of γ_1_34.5 has been deleted but retains two copies of the γ_1_34.5 C-terminal domain. ΔN146 has been shown to inhibit translational shutdown, overall contributing to enhanced replication in a number of tumor cells lines and enhanced therapeutic efficacy in 4T1 syngeneic tumor models as compared to first-generation oHSV [125]. The oncolytic virus Δ68H-6 contains a deletion of amino acids 68–87 of γ_1_34.5 along with a deletion of ICP6 [126]. This region binds to Beclin 1 to prevent autophagy [13] but also overlaps with regions on γ_1_34.5 needed for viral egress and TBK-1 binding [16,127]. Δ68H-6 was shown to extend the survival time in a U87 glioma mouse xenograft model as compared to HSV1716 [126].

## 6. Improving Antitumor Immunity

It is now well recognized that in addition to tumor-selective replication, robust immune activation is critical for therapeutic efficacy of oHSV. In addition to ISGs to directly combat infection, IFN also stimulates the production of cytokines to recruit Dendritic Cells (DCs), Macrophages, and T lymphocytes to a site of infection. DCs and Macrophages are Antigen Presenting Cells (APCs) that phagocytose malignant or infected cells and process antigen peptides for presentation to B and T lymphocytes for activation [128].

To establish an immune-suppressed environment, the γ_1_34.5 protein can inhibit DC maturation through the abrogation of TBK-1 and IKK activation of IFN and NF-κB, impairing antigen presentation and T lymphocyte activation during HSV infection [18,129]. Similarly, the malignant inhibition of cGAS/STING signaling through epigenetic silencing, mutant p53, or HER2 results in the simultaneous suppression of T lymphocyte infiltration in in vivo tumor models [34,35,78]. The activation of tumor endogenous STING has been identified as an important component for inducing antitumor immune responses [130]. The malignant suppression of cGAS/STING provides an opportunity for oncolytic replication, but the absence of STING signaling can restrict immunogenic cell death that is associated with robust activation of the immune response [130,131,132]. In addition to silencing of antitumor immune activation at the molecular level through cGAS/STING inhibition, malignancy has developed mechanisms of immunosuppression at the cellular level through the overexpression of immune checkpoint molecules (PD1, PDL1, and CTLA4) that normally act to prevent overactive T lymphocyte responses [133]. While this immunosuppressed environment provides an opportunity for oHSV replication, hampered APC activation and T lymphocyte target recognition can lead to ineffective antitumor responses [134]. Thus, many oHSV have been engineered to express cellular cytokines and chemokines to circumvent these crippled innate immune signaling pathways and enhance immune cell stimulation (Table 1).

DCs are major producers of IL-12, which is a cytokine that is capable of enhancing the activation and proliferation of cytotoxic T lymphocytes and NK cells, resulting in increased IFN-γ, a Type II IFN, production from these cell types [135]. IFN-γ can upregulate MHC I expression in tumor cells and transform immunosuppressive M2 macrophage into the immunostimulatory M1 subtype [136]. Although the systemic administration of IL-12 results in serious adverse side effects in humans [136], several oHSV, including M032, encode IL-12 for localized tumor expression (Table 1). M032 has recently entered a Phase I clinical trial for the treatment of glioma [97]. GM-CSF is another cytokine frequently expressed in oHSV. GM-CSF enhances the activation, proliferation, and recruitment of APCs from bone marrow [137]. In addition to α expression of Us11, T-VEC encodes two copies of GM-CSF in an effort to improve APC activation and antigen presentation in an environment replete with tumor antigens from viral oncolysis [100]. APCs ultimately prime cytotoxic T lymphocytes, which are major contributors to the antitumor response [138], and numerous studies have identified positive correlations between infiltrating cytotoxic T lymphocytes and improved therapeutic outcomes [134].

To further examine the immune effectors driving antitumor responses with T-VEC monotherapy, a recently completed Phase II clinical trial analyzed the correlates between immune cell infiltration and objective response rate in individuals with stage IIIB-IVM1a melanoma [139]. The study recorded an objective response rate of 28% and a complete response rate of 14%, which is on par with previous T-VEC melanoma trials [139,140,141]. Intriguingly, there was a 2.4-fold increase in T lymphocyte infiltration in non-injected lesions 6 weeks after initial treatment yet, CD4+ or CD8+ T lymphocyte infiltration did not correlate with the objective response rate [139]. Notably, activated T lymphocytes secreting IFN-γ can upregulate PDL1 expression in the epithelium as a negative feedback mechanism to control hyperactivation [133]. Exploratory analysis revealed detectable PDL1 expression in non-injected lesions of 10/50 patients at baseline, and this increased to 28/50 patients after 6 weeks of T-VEC treatment. This is suggestive of adaptive resistance to T lymphocyte infiltration in which malignancy upregulates immune checkpoints in non-injected lesions of patients treated with T-VEC [139]. This study reinforces combination therapy approaches using immune checkpoint inhibitors (anti-PD1/PDL1) and provides initial evidence to explain the synergistic effects seen when combining T-VEC with checkpoint blockade (Phase 1b: T-VEC in combination with anti-PD1 for advanced melanoma: objective response rate 62%, complete response rate 33%) [142]. In addition, these trials make clinical stage oHSVs such as RP2, ONCR-177, and VG161 particularly exciting as these viruses, among other modifications, encode PD1 antagonist peptides or anti-PD1/CTLA4 monoclonal antibodies, possibly providing a unique angle to combat malignancy in the tumor microenvironment (Table 1).

## 7. Perspectives

The clinical efficacy of T-VEC has led to the explosive growth of γ_1_34.5-attenuated oHSV development and subsequent clinical trials. While this growth will ultimately be beneficial for cancer therapy, much remains to be improved, especially for patients with advanced disease. A multitude of possible cancer genotypes and phenotypes yields a myriad of possible mechanisms of action for individual oHSVs to exert therapeutic effect. The recent studies regarding the malignant abrogation of IFN responses reviewed here provide a glimpse into the molecular and cellular determinants of tumor susceptibility to γ_1_34.5-attenuated oHSV. Further understanding of the complex layer of virus–host interactions underlying tumor selective replication and antitumor immunity will be critical for development of the next generation of oHSV as well as providing essential information for better patient stratification criteria to improve clinical outcomes.
